# Swinholide J, a Potent Cytotoxin from the Marine Sponge *Theonella swinhoei*

**DOI:** 10.3390/md9061133

**Published:** 2011-06-22

**Authors:** Simona De Marino, Carmen Festa, Maria Valeria D’Auria, Thierry Cresteil, Cecile Debitus, Angela Zampella

**Affiliations:** 1 Department of Chemistry of Natural Compounds, University of Naples, “Federico II”, via D. Montesano 49, 80131 Napoli, Italy; E-Mails: sidemari@unina.it (S.D.M.); carmen.festa@unina.it (C.F.); madauria@unina.it (M.V.D.); 2 Natural Substances Institute, la Terrasse Street, 91198 Gif sur Yvette Cedex, France; E-Mail: cresteil@icsn.cnrs-gif.fr; 3 Polynesian Research Center on Island Biodiversity, UMR 7138 CNRS, B.P. 529, 98713 Papeete, Tahiti, French Polynesia, France; E-Mail: cecile.debitus@ird.fr

**Keywords:** marine cytotoxin, swinholide J, *Theonella swinhoei*

## Abstract

In our ongoing search for new pharmacologically active leads from Solomon organisms, we have examined the sponge *Theonella swinhoei*. Herein we report the isolation and structure elucidation of swinholide A (**1**) and one new macrolide, swinholide J (**2**). Swinholide J is an unprecedented asymmetric 44-membered dilactone with an epoxide functionality in half of the molecule. The structural determination was based on extensive interpretation of high-field NMR spectra and HRESIMS data. Swinholide J displayed potent *in vitro* cytotoxicity against KB cells (human nasopharynx cancer) with an IC_50_ value of 6 nM.

## 1. Introduction

*Theonella* sponges represent an extraordinary source of bioactive secondary metabolites, particularly peptides and macrolides. Swinholide A (**1**) was the first symmetric 44-membered macrolide to be isolated from the Red Sea marine sponge *Theonella swinhoei* [1], and then demonstrated as product of biochemistry of symbiontic microorganisms [2]. The structure was first assigned as a monomer, revised later to a symmetric cyclic dimer [3], followed by determination of its stereochemistry [4–6]. Swinholide A (**1**) displays impressive biological properties including antifungal activity and potent cytotoxicity against a number of tumor cells. Its mechanism of action has been clarified in detail and its activity has been attributed to its ability to dimerize actin and disrupt the actin cytoskeleton [7–9]. Today, swinholide A is one of the better-characterized membrane permeable and specific inhibitors of actin filament networks and is actively used in cell biology studies [10].

Several derivatives of swinholide A were reported in the literature. They differ from the parent compound in the carbon backbone as in misakinolides [11–13] and hurghadolide [14] which feature a 40- and 42-membered dilactone structure, respectively, in the regiochemistry of the ring closure as in isoswinholide A [15], in the glycosidation, as in ankaraholides A and B [2], in the monomeric structure of preswinholide A [16], and in the different symmetric or asymmetric functionalization of the carbon backbone as in swinholides B (16′-demethyl) [15], C (29′-*O*-demethyl) [15], D (15′-*O*-demethyl) [17], E (6′-hydroxy) [17], F (2′-*Z* conformer) [17], G (20′-demethyl) [17], H (7,7′-*O*-dimethyl) [18] and I (26′-hydroxy) [14].

**Figure 1:**
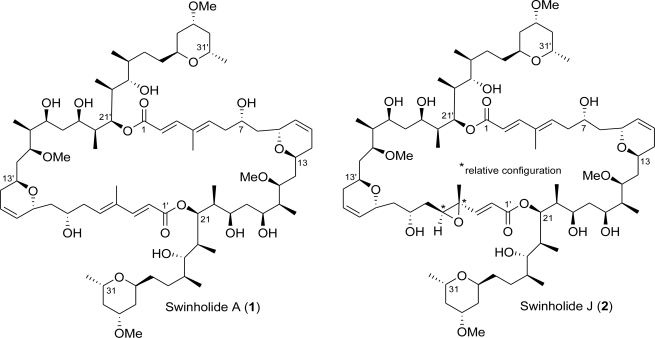
Swinholides A (**1**) and J (**2**) from *Theonella swinhoei*.

In our ongoing search for new pharmacologically active lead compounds from Solomon organisms [19–24], we have examined the sponge *Theonella swinhoei*. Separation of the cytotoxic fractions from the CHCl_3_ extract of a specimen of the marine sponge collected in Vangunu at the Solomon Islands, resulted in the identification of swinholide A (**1**) and of a new potently cytotoxic macrolide, swinholide J (**2**) (Figure 1).

## 2. Results and Discussion

Swinholide J (**2**) showed an intense ion peak at *m/z* 1427.9181 [(M + Na)^+^] in the HR ESIMS, 16 mass units higher than that observed for **1**, corresponding to one additional oxygen atom. As already reported for several swinholide derivatives [14,15,17], inspection of the ^1^H NMR spectrum clearly revealed an asymmetric dimeric nature for **2**. Notably, the ^1^H NMR spectrum of **2** showed seven resonances in the region 7.46–5.67 ppm (H-2, H-2′, H-3, H-3′, H-5, H-10/H-10′, H-11/H-11′), instead of resonances for five pairs of equivalent protons as in **1** (Table 1). Furthermore, in contrast to **1**, the ^1^H NMR spectrum of swinholide J (**2**) displayed two additional resonances at δ_H_ 3.22 [H-5′, dd (*J* = 4.3, 7.4 Hz)] and 1.42 (Me-4′, s). Also, the ^13^C NMR spectrum (Table 1), interpreted with the help of the HSQC and HMBC experiments, revealed the loss of symmetry in the dilactone skeleton of **2**. In the 152.4–115.9 ppm region, the ^13^C NMR spectrum of **2** showed eight resonances for olefinic carbons (nine methines, two of which corresponding to two pairs of equivalent carbons (C-10/C-10′ and C-11/C-11′) and one quaternary carbon, C-4), instead of six signals for six pairs of equivalent olefinic carbons as seen in **1**, and two resonances at δ_C_ 170.2 and 168.7 for the lactone carbons C-1 and C-1′, respectively. Moreover, in the region 55–80 ppm, two additional resonances respect to **1** were inferred from analysis of NMR data: one oxygen-bearing methine carbon (δ_C_ 64.6, δ_H_ 3.22, C-5′) and one oxygen-bearing quaternary carbon (δ_C_ 59.7, C-4′).

**Figure 2:**
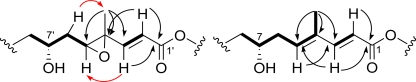
COSY/TOCSY connectivities (bold bonds), HMBC (black arrows) and ROE (red arrows) correlations for C-1/C-7 and C-1′/C-7′ partial structures of swinholide J (**2**).

All these data clearly suggested a perturbation in the diene moiety of one half of the molecule with the introduction of an epoxide functionality. Extensive study of COSY, HSQC, and HMBC spectra allowed us to establish the presence in the molecule of one half identical to that of symmetric swinholide A (Figure 2). A conjugated double bond in the other half (C-1′–C-3′) was inferred by COSY correlation between proton signals at δ_H_ 6.14 (d, *J* = 15.7 Hz) and 6.82 (d, *J* = 15.7 Hz) and HMBC cross-peaks H-2′/C-1′ and H-3′/C-1′. The two additional oxygen-bearing carbons were placed at C-4′ and C-5′, respectively, on the basis of HMBC correlations from methyl protons at δ_H_ 1.42 (3H, s, Me-4′) to C-3′, C-4′ and C-5′. Definitive confirmation of the proposed structure for swinholide J (**2**), derived from 2D-HOHAHA analysis, showed correlations starting from H-5′ (δ_H_ 3.22) to H-9′ (δ_H_ 4.48).

As shown in Table 1, the presence of an epoxy-functionality at C-4′/C-5′ positions at one side of the molecule caused twinning of most of the ^1^H and ^13^C NMR resonances of the nuclei belonging to dilactone ring skeleton, without any effect on the signals of the side chain.

The large coupling constant (15.7 Hz) between two vinyl protons H-2′ and H-3′ revealed the *E*-configuration of Δ2′-double bond whereas ROE correlations Me-4′/H-6′ and H-5′/H-3′ allowed us to established a relative configuration around the epoxide moiety as depicted in Figure 2. The stereochemistry of all the remaining stereocenters of swinholide J (**2**) is suggested to be the same as the parent swinholide A (**1**) on the basis of the similarity in their chemical shift and in the coupling constant values.

Swinholide J (**2**) was isolated as a very minor component with respect to the parent compound swinholide A (**1**) (relative composition of **1**/**2** in the sponge extract being 15:1 whereby the % yield was calculated based on the wet weight of the CHCl_3_ extract, swinholide A 0.55% and swinholide J 0.038%). On this basis, swinholide J (**2**) could be considered to be artifact arising from the extraction and isolation procedures utilized. However, careful HPLC and ^1^H NMR (700 MHz) analyses of all swinholide-containing fractions did not show the presence of other isomeric mono-epoxide or diepoxide derivatives that could be obtained through an abiotic radical oxidation of swinholide A (**1**). As such, swinholide J (**2**) is considered to be a new natural derivative of swinholide A (**1**) arising from a regio- and stereo-selective enzyme mediated oxidation of one of the two dienoate moieties in the parent compound.

Swinholide J (**2**) showed potent *in vitro* antiproliferative activity against KB cells, with an IC_50_ value of 6.7 nM, comparable to that of the parent compound (IC_50_ 1.2 nM against KB cells) [25].

## 3. Experimental Section

### 3.1. General Procedures

Specific rotations were measured on a Perkin-Elmer 243 B polarimeter. High-resolution ESI-MS spectra were performed with a Micromass QTOF Micromass spectrometer. ESI-MS experiments were performed on an Applied Biosystem API 2000 triple-quadrupole mass spectrometer. NMR spectra were obtained on Varian Inova 700 NMR spectrometer (^1^H at 700 MHz, ^13^C at 175 MHz, respectively) equipped with a Sun hardware, δ (ppm), *J* in Hz, spectra referred to CD_3_OH as internal standard (δ_H_ 3.31, δ_C_ 49.0). HPLC was performed using a Waters Model 510 pump equipped with Waters Rheodine injector and a differential refractometer, model 401.

Through-space ^1^H connectivities were evidenced using a ROESY experiment with mixing times of 200 ms.

Silica gel (200–400 mesh) from Macherey-Nagel Company was used for flash chromatography.

### 3.2. Sponge Material and Separation of Individual Macrolides

*Theonella swinhoei* (order Lithistida, family Theonellidae) was collected on the barrier reef of Vangunu Island, Solomon Islands, in July 2004. The samples were frozen immediately after collection and lyophilized to yield 207 g of dry mass. Taxonomic identification was performed by Dr. John Hooper of Queensland Museum, Brisbane, Australia, where specimen is deposited under the accession number G3122662.

The lyophilized material (207 g) was extracted with methanol (3 × 1.5 L) at room temperature and the crude methanolic extract was subjected to a modified Kupchan’s partitioning procedure as follows. The methanol extract was dissolved in a mixture of MeOH/H_2_O containing 10% H_2_O and partitioned against *n*-hexane (15.2 g). The water content (% v/v) of the MeOH extract was adjusted to 30% and partitioned against CHCl_3_ (5.8 g). The aqueous phase was concentrated to remove MeOH and then extracted with *n*-BuOH (6.0 g).

The CHCl_3_ extract (5.8 g) was chromatographed by silica gel MPLC using a solvent gradient system from CH_2_Cl_2_ to CH_2_Cl_2_:MeOH 1:1.

Fractions eluted with CH_2_Cl_2_:MeOH 95:5 (239 mg) were further purified by HPLC on a Nucleodur 100-5 C18 (5 μm; 7.8 mm i.d. × 250 mm) with MeOH:H_2_O (9:1) as eluent (flow rate 1 mL/min) to give 2.2 mg of swinholide J (**2**) (*t*_R_ = 7.0 min) and 31.7 mg of swinholide A (**1**) (*t*_R_ = 7.4 min).

Biological evaluation. The antiproliferative activity of swinholide A and J was determined on KB (nasopharyngeal epidermoid carcinoma) as previously reported [26].

### 3.3. Characteristic Data for Each Compound

**Swinholide A** (**1**): light yellow solid; [α]_D_^23^ +16.1 (*c* 0.12, MeOH); ^1^H and ^13^C NMR data in CD_3_OD given in Table 1; ESIMS: *m*/*z* 1411.9 [M + Na]^+^. HRMS (ESI): calcd for C_78_H_132_NaO_20_: 1411.9210; found 1411.9257 [M + Na]^+^.

**Swinholide J** (**2**): light yellow solid; [α]_D_^23^ −11.7 (*c* 0.14, MeOH); ^1^H and ^13^C NMR data in CD_3_OD given in Table 1; ESIMS: *m*/*z* 1427.9 [M + Na]^+^. HRMS (ESI): calcd for C_78_H_132_NaO_21_: 1427.9159; found 1427.9181 [M + Na]^+^.

## 4. Conclusions

In this paper we report the isolation and the structural characterization of a new swinholide congener, swinholide J endowed with potent cytotoxic activity from the marine sponge *Theonella swinhoei*. The structure was determined by extensive application of 2D NMR techniques. The discovery of swinholide J reaffirms the utility of examining marine sponge for identifying novel potential antitumor lead compounds.

## Figures and Tables

**Table 1:** NMR data (700 MHz, CD_3_OD) for swinholides A (**1**) and J (**2**) (δ in ppm, *J* in Hz).

		**1^a^**		**2^a^**		
**Position**	**Type**	**δ_H_/δ_H′_ (*J* in Hz)**	**δ_C_/δ_C′_**	**δ_H_/δ_H′_ (*J* in Hz)**	**δ_C_/δ_C′_**	**HMBC^b^**
1/1′	C	-	170.6	-	170.2/168.7	
2/2′	CH	5.84 d (15.7)	115.6	5.90 d (15.6)/6.14 d (15.7)	115.9/122.2	C1, C4
						C1′, C4′
3/3′	CH	7.43 d (15.7)	152.3	7.46 d (15.6)/6.82 d (15.7)	152.0/152.4	C1, C5
						C1′
4/4′	C	-	135.5	-	135.5/59.7	
4/4′-Me	CH_3_	1.77 s	12.4	1.85 s/1.42 s	12.7/15.5	C3, C4, C5
						C3′, C4′, C5′
5/5′	CH	6.14 t (7.3)	140.5	6.16 t (7.2)/3.22 dd (4.3, 7.4)	140.6/64.6	C3, 4-Me
6/6′	CH_2_	2.40 t (6.9)	38.8	2.44 m/1.81 m, 1.63 m	38.7/37.9	C4, C5, C7, C8
7/7′	CH	4.02 m	68.1	3.99 ovl/4.12 m	68.2/66.5	
8/8′	CH_2_	1.28 m	41.0	1.87 m, 1.37 m/	41.8/41.6	
		1.76 m		1.78 m, 1.37 m		
9/9′	CH	4.47 br d (10.5)	70.5	4.48 br d (10.2)	70.4	
10/10′	CH	5.65 dd (1.8, 10.5)	130.9	5.67 br d (10.3)	130.8	
11/11′	CH	5.81 m	124.9	5.83 m	124.8	
12/12′	CH_2_	1.94 m	32.2	1.96 m	32.2	
13/13′	CH	3.49 m	65.3	3.54 m	65.4	
14/14′	CH_2_	1.58 m	37.2	1.58 m	36.9	
		1.77 m		1.82 m		
15/15′	CH	3.76 m	78.2	3.76 m/3.83 m	78.4/77.9	
15/15′-OMe	CH_3_	3.32 s	56.7	3.34 s/3.35 s	56.9/57.2	C15/C15′
16/16′	CH	1.52 m	43.8	1.55 m	43.3	
16/16′-Me	CH_3_	0.83 d (6.7)	8.8	0.83 d (7.0)/0.84 d (7.0)	9.2	C15, C16, C17
						C15′, C16′, C17′
17/17′	CH	3.61 m	73.2	3.60 m	73.2	
18/18′	CH_2_	1.63 m	39.0	1.61 m	39.2	
		1.74 m		1.75 m		
19/19′	CH	3.97 ovl	70.1	3.97 ovl/3.88 m	70.0/70.1	
20/20′	CH	1.94 m	39.4	1.94 m	39.4	
20/20′-Me	CH_3_	0.91 d (7.0)	8.9	0.90 d (7.1)/0.91 d (7.1)	9.1	
21/21′	CH	5.46 d (10.5)	75.6	5.45 t (10.3)/5.46 t (10.3)	75.7/76.3	
22/22′	CH	1.98 m	37.9	1.97 m	38.0	
22/22′-Me	CH_3_	0.94 d (6.9)	9.6	0.92 d (7.0)/0.93 d (7.0)	9.7	C21, C22, C23
						C21′, C22′, C23′
23/23′	CH	3.11 dd (1.8, 9.5)	77.2	3.10 m	77.3	
24/24′	CH	1.70 m	34.4	1.70 m	34.5	
24/24′-Me	CH_3_	0.98 d (6.7)	17.7	0.97 d (6.7)/0.98 d (6.7)	17.9	C23, C24, C25
						C23′, C24′, C25′
25/25′	CH_2_	1.24 m	25.1	1.23 m, 1.41 m	25.1	
		1.42 m				
26/26′	CH_2_	1.27 m	29.7	1.28 m, 1.94 m	29.8	
		1.94 m				
27/27′	CH	3.99 ovl	72.7	3.98 ovl	72.8	
28/28′	CH_2_	1.52 m	35.8	1.52 m	35.8	
		1.87 br d (12.8)		1.87 br d (12.5)		
29/29′	CH	3.61 m	74.2	3.61 m	74.3	C29-OMe
						C29′-OMe
29/29′-OMe	CH_3_	3.34 s	55.3	3.34 s	55.3	C29
						C29′
30/30′	CH_2_	1.09 dd (10.4, 12.6)	39.7	1.09 dd (10.4, 12.6)	39.7	
		2.01 br d (12.6)		2.02 br d (12.6)		
31/31′	CH	3.74 m	65.7	3.74 m	65.8	
31/31′-Me	CH_3_	1.19 d (6.2)	21.8	1.19 d (6.2)	21.8	

a Data from COSY, HSQC, and HMBC experiments;

b HMBC correlations, optimized for 6 Hz, are from proton(s) stated to the indicated carbon.
